# Knockdown of KLF5 suppresses hypoxia-induced resistance to cisplatin in NSCLC cells by regulating HIF-1α-dependent glycolysis through inactivation of the PI3K/Akt/mTOR pathway

**DOI:** 10.1186/s12967-018-1543-2

**Published:** 2018-06-14

**Authors:** Tianxiao Gong, Liuqing Cui, Haili Wang, Haoxun Wang, Na Han

**Affiliations:** 1 Department of Oncology, The Second Affiliated Hospital of Zhengzhou University, No. 2 Jingba Road, Zhengzhou, 450014 People’s Republic of China; 2 College of Bioengineering, Henan University of Technology, Lianhua Street, Zhengzhou, 450001 People’s Republic of China

**Keywords:** KLF5, Hypoxia, DDP resistance, Glycolysis, The PI3K/Akt/mTOR pathway, NSCLC

## Abstract

**Background:**

Hypoxia-mediated chemoresistance has been regarded as an important obstacle in the development of cancer treatment. Knockdown of krüppel-like factor 5 (KLF5) was reported to inhibit hypoxia-induced cell survival and promote cell apoptosis in non-small cell lung cancer (NSCLC) cells via direct regulation of hypoxia inducible factor-1α (HIF-1α) expression. However, the roles of KLF5 in the development of hypoxia-induced cisplatin (DDP) resistance and its underlying mechanism in NSCLC cells remain to be further elucidated.

**Methods:**

Western blot was performed to determine the protein levels of KLF5, P-glycoprotein (P-gp) and HIF-1α in treated NSCLC cells. Cell survival was examined by MTT assay. The effect of KLF5 knockdown on hypoxia-induced glycolysis was assessed by measuring glucose consumption and lactate production. The effect of KLF5 knockdown on the phosphoinositide 3-kinase (PI3K)/protein kinase B (Akt)/mammalian target of rapamycin (mTOR) pathway was analyzed by western blot.

**Results:**

Hypoxia upregulated the expression of KLF5 in NSCLC cells. KLF5 knockdown suppressed hypoxia-induced DDP resistance in NSCLC cells, as demonstrated by the increased cytotoxic effects of DDP and reduced P-gp expression in NSCLC cells in hypoxia. Moreover, KLF5 knockdown inhibited hypoxia-induced HIF-1α expression and glycolysis, and KLF5 knockdown suppressed hypoxia-induced DDP resistance by inhibiting HIF-1α-dependent glycolysis in NSCLC cells. Furthermore, KLF5 knockdown suppressed hypoxia-induced activation of the PI3K/Akt/mTOR pathway in NSCLC cells and KLF5 overexpression promoted hypoxia-induced DDP resistance in NSCLC cells through activation of the PI3K/Akt/mTOR pathway.

**Conclusions:**

KLF5 knockdown could suppress hypoxia-induced DDP resistance, and its mechanism may be due to the inhibition of HIF-1α-dependent glycolysis via inactivation of the PI3K/Akt/mTOR pathway.

## Background

Lung cancer, one of the most common cancer types in women and men, is currently the predominant cause of cancer-related deaths worldwide, resulting in more than one million deaths worldwide annually [1]. Non-small cell lung cancer (NSCLC) comprises almost 85% of all lung cancer cases [2]. Despite the considerable improvements in early diagnosis and therapeutic approaches of NSCLC patients, the prognosis is still unsatisfactory, with the total 5-year survival rate less than 15% [3]. Platinum-based chemotherapy is an important adjuvant therapeutic strategy for NSCLC patients [4]. As the most widely used platinum compound, cisplatin (DDP) is the first-line chemotherapeutic agent for the treatment of NSCLC and can induce cancer cell cycle arrest and apoptotic death [5]. However, its efficacy is often impaired due to the development of chemoresistance, which accounts for the major chemotherapy failure in both resectable and advanced NSCLC [6]. Therefore, elucidating the molecular mechanism involved in DDP resistance of NSCLC and identifying novel molecular-targeted therapeutic approaches to overcome DDP resistance are desperately needed.

The mechanism of chemoresistance is highly complicated, in which a hypoxic microenvironment potentially plays a critical role [7]. Hypoxia is a common phenomenon in most human solid tumors and has the potential to affect various physiological and pathological processes, such as proliferation, apoptosis, angiogenesis, and therapy resistance [8]. Increasing clinical and experimental studies have documented that hypoxia contributes to resistance to anticancer drugs in diverse cancer cells by promoting malignant phenotypes [9, 10]. Accordingly, hypoxia-mediated chemoresistance has been regarded as an important obstacle in the development of cancer treatment [11]. Hypoxia inducible factor-1α (HIF-1α) is an important regulator of transcription in response to hypoxia, which activates the expression of more than 60 target genes involved in cell proliferation, apoptosis, and glycolysis [12]. It is believed that HIF-1α-induced glycolysis plays a critical role in promoting chemoresistance of NSCLC cells [13]. Recent studies have also reported the involvement of glycolysis with chemoresistance [14]. Therefore, inhibiting HIF-1α-induced glycolysis may be a potential approach for the reversal of hypoxia-induced chemoresistance.

Krüppel-like factor 5 (KLF5, also known as BTEB2), a member of the KLF family, is characterized by containing a highly conserved C-terminal and three independent c2h2 zinc fingers [15]. KLF5 binds to the GC-rich DNA sequence in the promoters of target genes using its three zinc finger domains [16]. As a DNA-binding transcriptional factor, KLF5 has been shown to have essential roles in multiple cellular processes, including tumor progression, cell proliferation, differentiation and apoptosis, in different human cancers [17]. More importantly, KLF5 is well acknowledged to be regulated by hypoxia in tumors and acts as an upstream regulator of HIF-1α [18]. A previous study reported that KLF5 knockdown inhibited hypoxia-induced cell survival and promoted cell apoptosis in NSCLC cells via direct regulation of HIF-1α expression [19]. However, the roles of KLF5 in the development of hypoxia-induced DDP resistance and its underlying mechanism in NSCLC cells remain to be further elucidated.

In the present study, we demonstrated that KLF5 contributed to hypoxia-induced DDP resistance in NSCLC cells and the mechanism of KLF5 was elucidated.

## Methods

### Cell lines and hypoxic incubation

NSCLC cell lines H1299 and A549 were purchased from American Type Culture Collection (ATCC, Manassas, VA, USA). These cells were cultured in RPMI-1640 medium (Gibco, Grand Island, NY, USA) containing 10% fetal bovine serum (FBS; Hyclone, Logan, UT, USA), 100 U/mL penicillin (Thermo Scientific, Rockford, IL, USA) and 100 μg/mL streptomycin (Thermo Scientific) in a humidified incubator at 37 °C with 21% O_2_ and 5% CO_2_ for normoxic condition. Cells were maintained in a humidified atmosphere at 37 °C under 94% N_2_, 1% O_2_ and 5% CO_2_ in the automated Xvivo system G300CL (BioSpherix, Lacona, NY, USA) for hypoxic condition.

### Cell transfection

siRNAs specifically against KLF5 (si-KLF5#1 and si-KLF5#2), siRNA scrambled control (si-NC), pcDNA-KLF5, pcDNA-HIF-1α, and pcDNA empty vector (Vector) were purchased from GenePharma (Shanghai, China). Cells were plated in 6-well plates at a density of 2.5 × 10^5^ cells/well and grown to 80% confluence. Subsequently, cell transfection with these oligonucleotides or plasmids was performed using Lipofectamine 2000 (Invitrogen, Carlsbad, CA, USA). At 48 h post-transfection, cells were harvested for further analysis.

### Western blot analysis

Total protein was extracted from cultured cells in RIPA lysis buffer (Beyotime, Beijing, China). Protein concentration of the cell extracts was measured by BCA Protein Assay Kit (Pierce, Rockford, IL, USA). Approximately 50 μg protein samples in each lane were separated by 12% sodium dodecyl sulfate-polyacrylamide gel electrophoresis (SDS-PAGE), followed by being transferred onto PVDF membrane (EMD Millipore, Billerica, MA, USA). Following blocking with 5% nonfat dry milk in TBST buffer (20 mM Tris–HCl, pH 7.4, 150 mM NaCl and 0.1% Tween 20) for 1 h, the membranes were incubated at 4 °C overnight with the primary antibodies against KLF5, P-glycoprotein (P-gp), HIF-1α, mammalian target of rapamycin (mTOR), phosphoinositide 3-kinase (PI3K), protein kinase B (Akt), phosphorylated-Akt (p-Akt) and β-actin (all from Abcam, Cambridge, MA, USA) and then incubated with horseradish peroxidase (HRP)-conjugated secondary antibody immunoglobulin G (IgG) (Santa Cruz Biotechnology Inc., Santa Cruz, CA, USA) at room temperature for 1 h. The blots were developed by enhanced chemiluminescence reagent (GE Healthcare, Piscataway, NJ, USA).

### Cell survival assay

A549 and H1299 cells were seeded into 96-well culture plates at a density of 5 × 10^3^ cells/well and transfected with or without si-KLF5 or si-NC, pcDNA-KLF5 or Vector. At 48 h post-transfection, various concentrations of DDP (0, 5, 10, 15, 20, 25, 30, 35, and 40 μM) were added to each well and the cells were incubated in a humidified incubator under a normoxic or hypoxic condition for another 24 h. Subsequently, 20 μL of 5 mg/mL 3-(4,5-dimethylthiazol-2-yl)-2,5-diphenyltetrazolium bromide (MTT; Sigma, St. Louis, MO, USA) was added to each well and incubated for another 4 h. Then the supernatant was discarded and 150 μL of dimethyl sulfoxide (DMSO, Sigma) was added to dissolve the dark blue crystal. Absorbance of each well was measured at 490 nm using a microplate reader (Bio-Rad, Hercules, CA, USA).

### Measurement of glucose uptake and lactate

A549 and H1299 cells were seeded into 6-well plates and transfected with or without si-KLF5 si-NC, pcDNA-KLF5 or Vector, followed by incubation under a normoxic or hypoxic condition. The culture supernatant of cells was collected after transfection for 48 h and the amount of glucose and lactate in the supernatant was measured using a glucose uptake colorimetric assay kit (Sigma) and a Lactic Acid assay kit (KeyGen, Nanjing, China), respectively.

### Statistical analysis

All results were expressed as the mean ± standard deviation (SD). Statistical analysis was performed using SPSS statistical software (version 18.0, SPSS, Inc., Chicago, IL, USA). The statistical differences between groups were determined by the two-tailed unpaired Student’s *t* test. *P* < 0.05 was considered to indicate a statistically significance.

## Results

### Hypoxia upregulated the expression of KLF5 in NSCLC cells

**Fig. 1:**
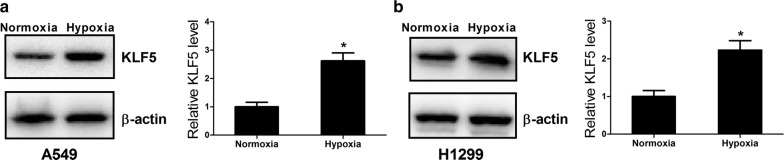
Hypoxia upregulated the expression of KLF5 in NSCLC cells. Western blot was performed to detect the protein level of KLF5 in A549 (**a**) and H1299 (**b**) cells under a normoxic or hypoxic condition. *P < 0.05

To determine the effect of hypoxia on the expression of KLF5 in NSCLC cells, we examined the protein level of KLF5 in A549 and H1299 cells exposed to hypoxia by western blot. As shown in Fig. 1a and b, KLF5 level was significantly higher in A549 and H1299 cells under hypoxia as compared with that under normoxia, indicating that hypoxia induced the upregulation of KLF5 in NSCLC cells.

### KLF5 knockdown suppressed hypoxia-induced DDP resistance in NSCLC cells

**Fig. 2:**
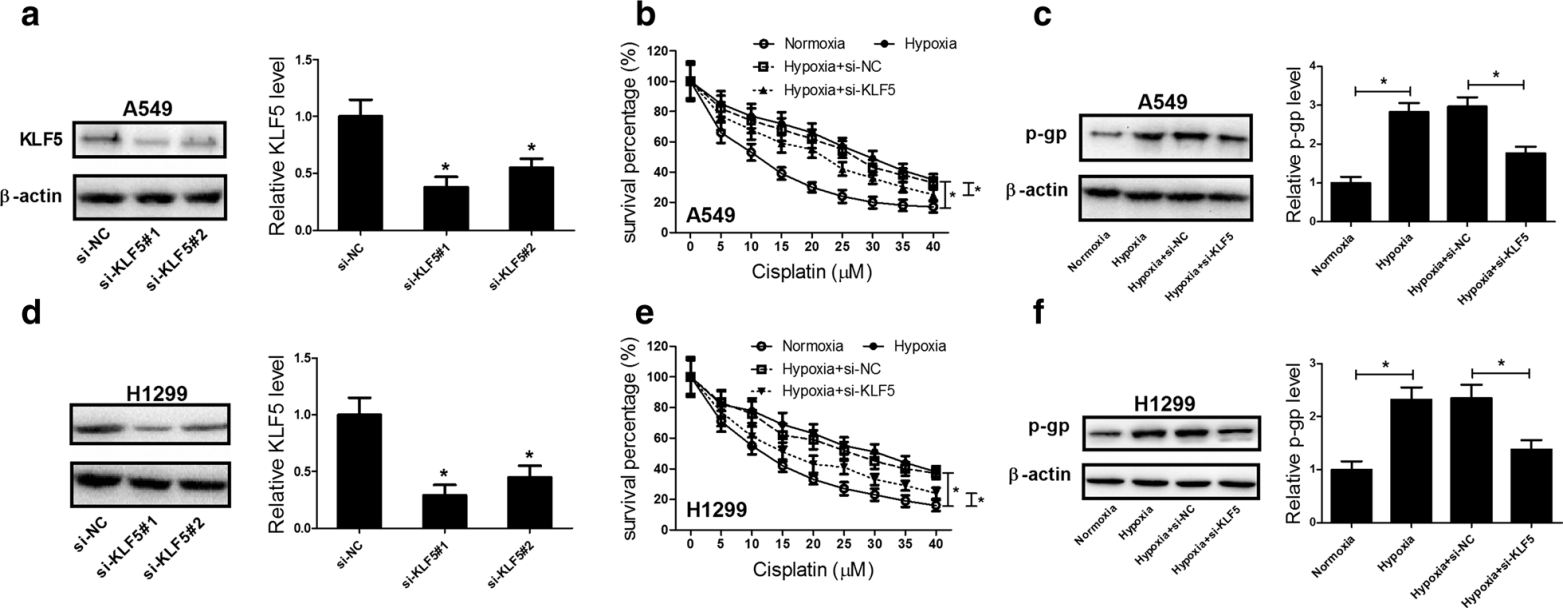
KLF5 knockdown suppressed hypoxia-induced DDP resistance in NSCLC cells. **a**, **d** Western blot was conducted to evaluate the protein level of KLF5 in A549 and H1299 cells transfected with si-KLF5#1, si-KLF5#2, or si-NC. **b**, **e** MTT assay was applied to detect cell survival after A549 and H1299 cells were transfected with or without si-KLF5 or si-NC, followed by treatment with various concentrations of DDP (0, 5, 10, 15, 20, 25, 30, 35, and 40 μM) under a normoxic or hypoxic condition. **c**, **f** Western blot was performed to examine the protein level of P-gp in A549 and H1299 cells transfected with or without si-KLF5 or si-NC under a normoxic or hypoxic condition. *P < 0.05

To assess the role of KLF5 on hypoxia-induced DDP resistance in NSCLC cells, A549 and H1299 cells were transfected with si-KLF5#1, si-KLF5#2, or si-NC to study the loss-of-functions. Western blot analysis showed that KLF5 protein level was markedly reduced in A549 (Fig. 2a) and H1299 (Fig. 2d) cells after transfection with si-KLF5#1 or si-KLF5#2 compared with si-NC group. Notably, si-KLF5#1 (si-KLF5) exhibited a higher knockdown efficiency and thus was selected for further experiments. MTT assay demonstrated that cell survival percentage of A549 and H1299 cells treated with DDP under normoxia condition was dose-dependently reduced. In contrast, incubation in hypoxia remarkably abated the cytotoxic effects of DDP at all different doses, suggesting that hypoxia induced DDP resistance in NSCLC cells. However, KLF5 knockdown effectively overturned the cytotoxic effects of DDP on A549 (Fig. 2b) and H1299 (Fig. 2e) cells under a hypoxic condition versus si-NC group, indicating that KLF5 knockdown dramatically abolished hypoxia-induced DDP resistance in NSCLC cells. Consistently, the protein level of P-gp, which is known to be responsible for drug resistance of various tumors [20], was obviously increased in A549 (Fig. 2c) and H1299 (Fig. 2f) cells exposed to hypoxia, which was significantly attenuated by transfection of si-KLF5. Collectively, these results demonstrated that KLF5 knockdown suppressed hypoxia-induced DDP resistance in NSCLC cells.

### KLF5 knockdown inhibited hypoxia-induced HIF-1α expression and glycolysis in NSCLC cells

**Fig. 3:**
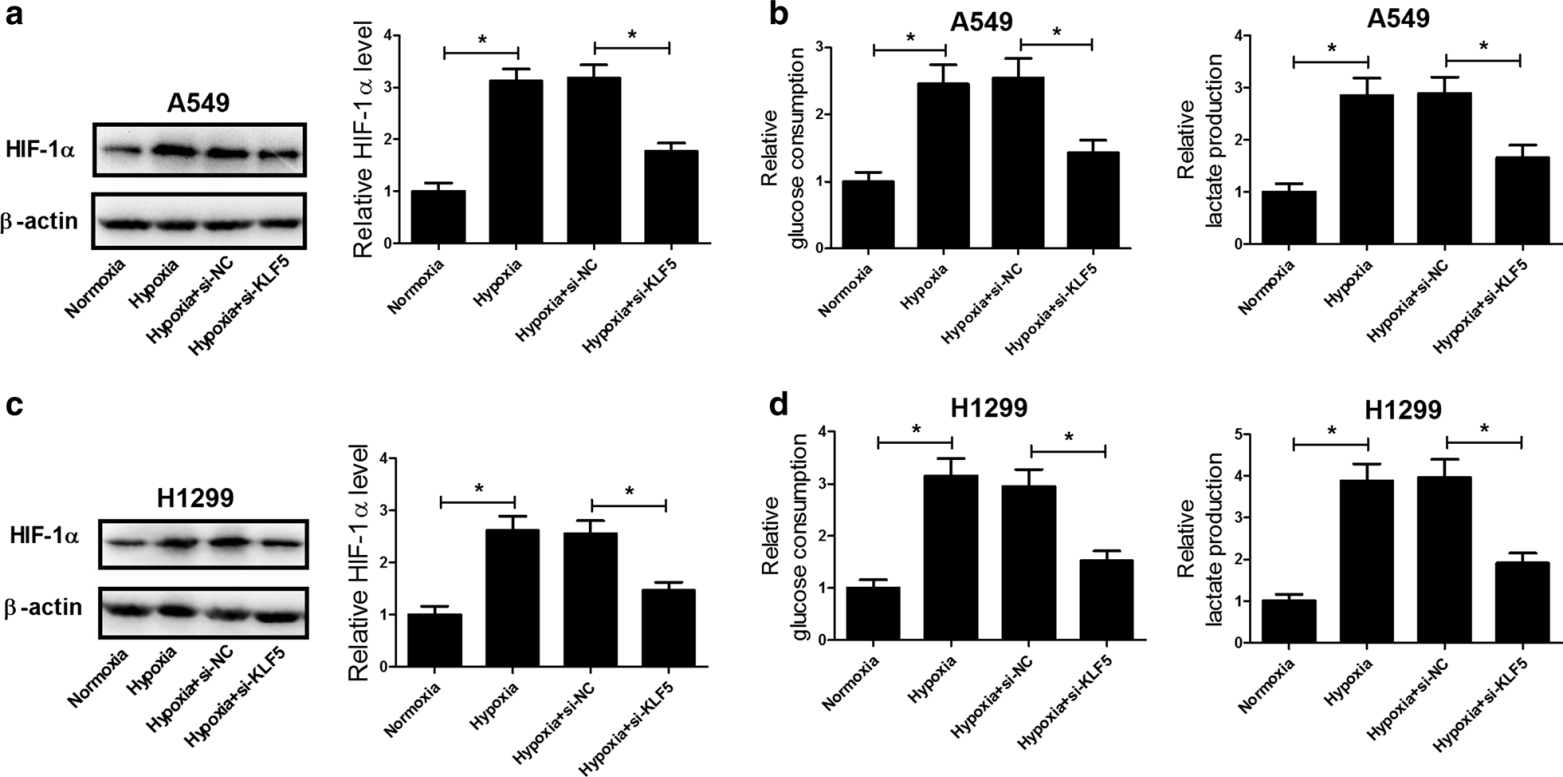
KLF5 knockdown inhibited hypoxia-induced HIF-1α expression and glycolysis in NSCLC cells. A549 and H1299 cells were transfected with or without si-KLF5 or si-NC and incubated under a normoxic or hypoxic condition for 48 h. Western blot analysis was performed to detect the protein level of HIF-1α in treated A549 (**a**) and H1299 (**c**) cells. Glucose consumption and lactic acid production of treated A549 (**b**) and H1299 (**d**) cells. *P < 0.05

It is believed that HIF-1α, a critical transcriptional factor in response to hypoxia, is closely related to the chemoresistance of many malignant tumors [21, 22]. We therefore analyzed the effect of KLF5 knockdown on the expression of HIF-1α in NSCLC cells under hypoxia by western blot and the results implied that hypoxia exposure enhanced the protein level of HIF-1α in A549 (Fig. 3a) and H1299 (Fig. 3c) cells, while KLF5 knockdown suppressed hypoxia-induced increase of HIF-1α expression. Additionally, increasing evidence has suggested that HIF-1α improves the glycolytic flux of cancer cells, which plays a critical role in promoting chemoresistance of NSCLC cells [23, 24]. Hence, the effect of KLF5 knockdown on hypoxia-induced glycolysis in NSCLC cells was investigated. As shown in Fig. 3b and d, hypoxia treatment led to obvious increases of glucose consumption and lactic acid production in A549 and H1299 cells compared with that in cells cultured in normoxia. However, the increased glucose consumption and lactic acid production under hypoxia were greatly reversed after transfection with si-KLF5. Together, these results indicated that KLF5 knockdown inhibited hypoxia-induced HIF-1α expression and glycolysis in NSCLC cells.

### KLF5 knockdown suppressed hypoxia-induced DDP resistance by inhibiting HIF-1α-dependent glycolysis in NSCLC cells

**Fig. 4:**
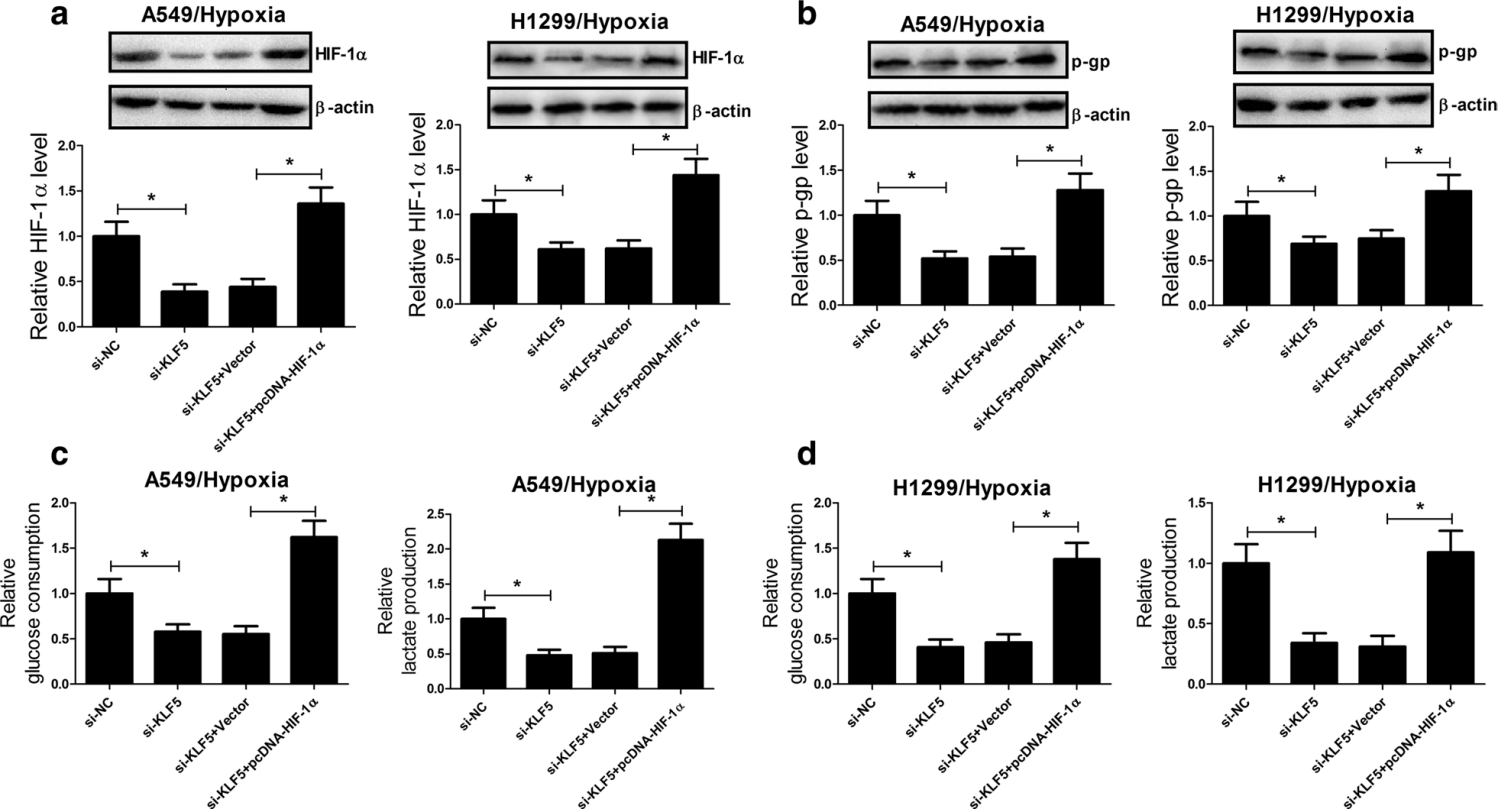
KLF5 knockdown suppressed hypoxia-induced DDP resistance by inhibiting HIF-1α-dependent glycolysis in NSCLC cells. A549 and H1299 cells were transfected with si-KLF5 or along with pcDNA-HIF-1α and incubated under a hypoxic condition. The protein levels of HIF-1α (**a**) and P-gp (**b**) in treated A549 and H1299 cells were determined by western blot. The glucose uptake (**c**) and lactate production (**d**) in treated A549 and H1299 cells were measured. *P < 0.05

To explore the molecular mechanism by which KLF5 knockdown suppressed hypoxia-induced DDP resistance in NSCLC, A549 and H1299 cells were transfected with si-KLF5 or along with pcDNA-HIF-1α and incubated under a hypoxic condition. It was observed that KLF5 knockdown significantly reduced the protein level of HIF-1α in A549 and H1299 cells under a hypoxic condition, while cotreatment with KLF5 knockdown and HIF-1α overexpression markedly recuperated HIF-1α expression (Fig. 4a). Additionally, the protein level of P-gp was conspicuously repressed by KLF5 deficiency in A549 and H1299 cells under a hypoxic condition, whereas ectopic expression of HIF-1α dramatically restored KLF5 knockdown-mediated inhibition of P-gp expression (Fig. 4b). We further assessed the effects of cotreatment with KLF5 knockdown and forced expression of HIF-1α on the glycolysis and the results demonstrated that KLF5 knockdown-mediated decrease of glucose uptake (Fig. 4c) and lactate production (Fig. 4d) in A549 and H1299 cells under hypoxia was notably recovered by HIF-1α overexpression. Therefore, we concluded that KLF5 knockdown suppressed hypoxia-induced DDP resistance by inhibiting HIF-1α-dependent glycolysis in NSCLC cells.

### KLF5 knockdown inhibited hypoxia-induced activation of the PI3K/Akt/mTOR pathway in NSCLC cells

**Fig. 5:**
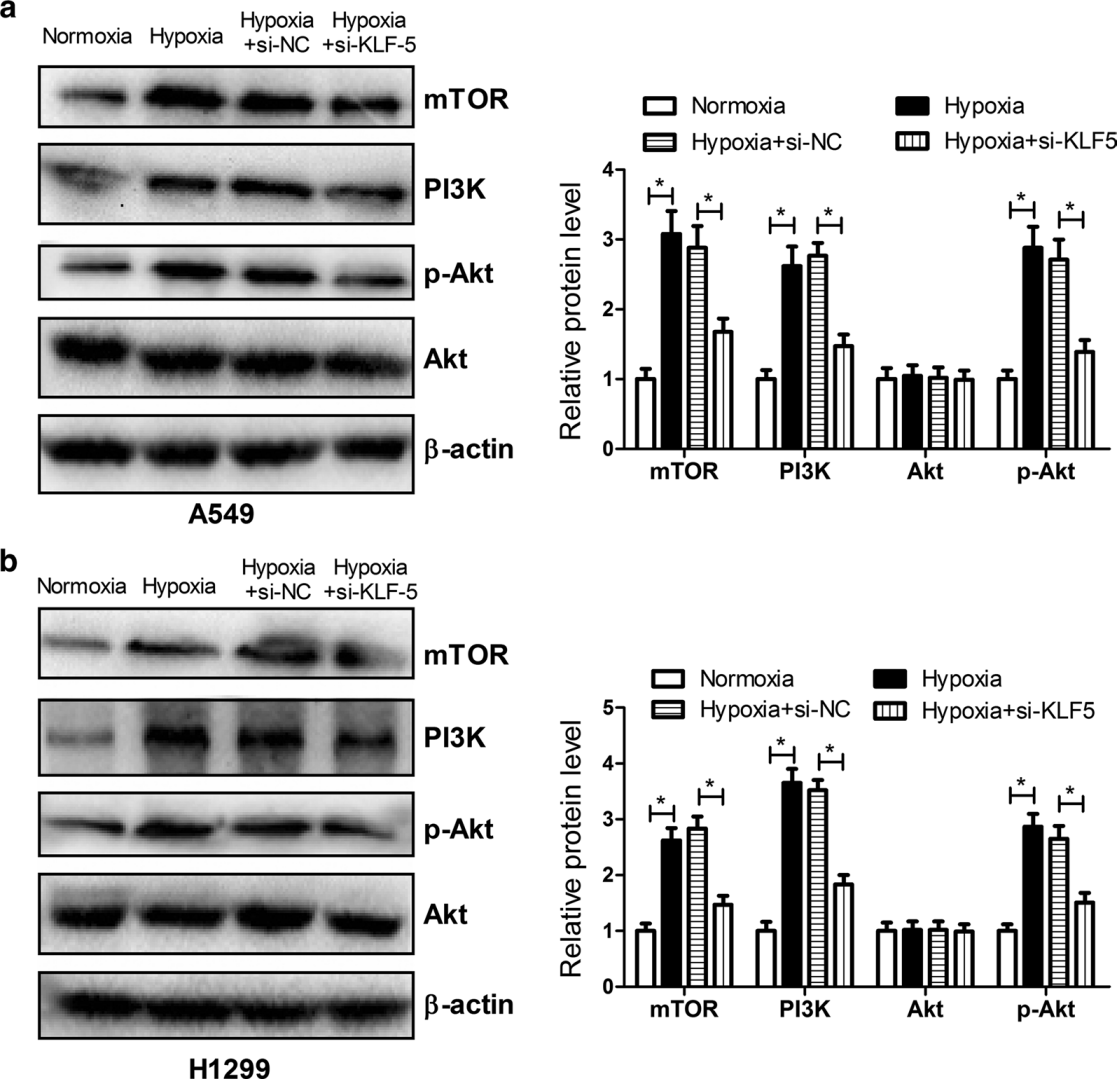
KLF5 knockdown inhibited hypoxia-induced activation of the PI3K/Akt/mTOR pathway in NSCLC cells. The protein levels of mTOR, PI3K, p-Akt, and Akt were determined by western blot after A549 (**a**) and H1299 (**b**) cells transfected with or without si-KLF5 or si-NC were incubated under a normoxic or hypoxic condition. *P < 0.05

It was previously documented that the PI3K/Akt/mTOR pathway was activated in response to hypoxia and mediated stabilization or activation of HIF-1α [25]. Moreover, activation of the PI3K/Akt pathway was reported to confer resistance to anti-cancer agents including DDP in lung cancer [26]. Therefore, we further analyzed the effect of KLF5 knockdown on the PI3K/Akt/mTOR pathway in NSCLC cells under a hypoxic condition. The results from western blot analysis proved that the protein levels of mTOR, PI3K, and p-Akt were strikingly facilitated in A549 (Fig. 5a) and H1299 (Fig. 5b) cells under a hypoxic condition compared with that under a normoxic condition, suggesting that hypoxia activated the PI3K/Akt/mTOR pathway in NSCLC cells. Nevertheless, KLF5 knockdown hindered the activation of the PI3K/Akt/mTOR pathway conferred by hypoxia in NSCLC cells.

### KLF5 overexpression promoted hypoxia-induced DDP resistance in NSCLC cells through activation of the PI3K/Akt/mTOR pathway

**Fig. 6:**
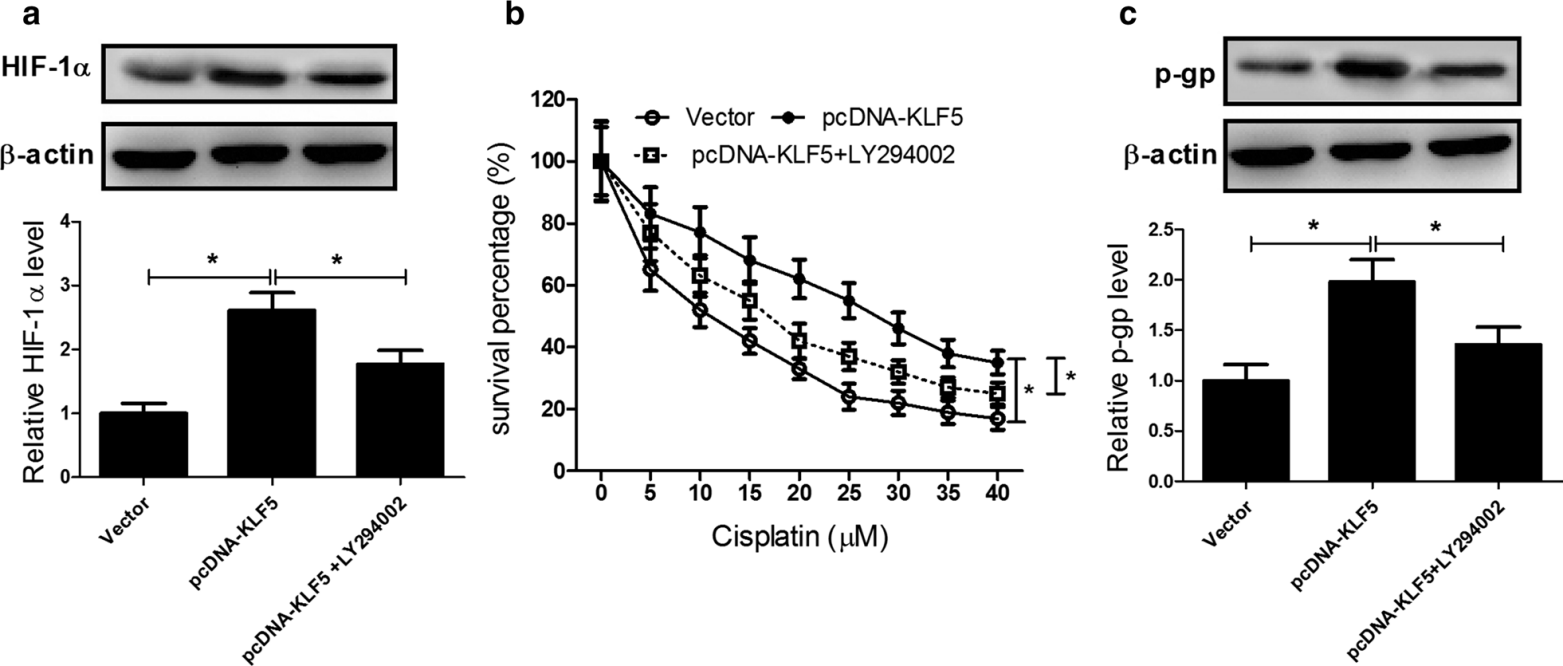
KLF5 overexpression promoted hypoxia-induced DDP resistance in NSCLC cells through activation of the PI3K/Akt/mTOR pathway. A549 cells were treated with Vector, pcDNA-KLF5 or combined with 10 μM LY294002 (a specific PI3K inhibitor) under hypoxia. The protein levels of HIF-1α (**a**) and P-gp (**c**) in treated A549 cells were detected by western blot. **b** MTT assay was conducted to assess cell survival in treated A549 cells. *P < 0.05

To address whether the PI3K/Akt/mTOR pathway was involved in KLF5-mediated regulation of hypoxia-induced DDP resistance in NSCLC cells, A549 cells were treated with Vector, pcDNA-KLF5 or combined with 10 μM LY294002 (a specific PI3K inhibitor) under hypoxia. As a result, elevated KLF5 expression apparently increased the protein level of HIF-1α in A549 cells under hypoxia, which was alleviated by LY294002 administration (Fig. 6a). Moreover, MTT assay showed that exogenous expression of KLF5 significantly relieved the cytotoxic effect of DDP on A549 cells under hypoxia when compared with that in Vector control group, while LY294002 dramatically reversed KLF5 overexpression-induced attenuation of the cytotoxic effect of DDP in A549 cells under hypoxia (Fig. 6b). Furthermore, P-gp level was effectively enhanced by KLF5 overexpression in A549 cells under hypoxia, while cotreatment with KLF5 overexpression and LY294002 dramatically suppressed the protein level of P-gp (Fig. 6c). Together, these data revealed that KLF5 overexpression promoted hypoxia-induced DDP resistance in NSCLC cells through activation of the PI3K/Akt/mTOR pathway.

## Discussion

It has been well documented that hypoxia-induced DDP resistance is one of the major obstacles in the chemotherapy of various solid tumors including NSCLC [27]. Therefore, it is urgently needed to figure out novel and effective sensitizers to overcome hypoxia-induced DDP resistance. In the present study, we described that KLF5 knockdown suppressed hypoxia-induced resistance to DDP in NSCLC cells and elucidated the underlying mechanism.

Accumulating evidence suggests that KLF5 has become the focus of the research due to its crucial regulatory functions associated with diverse functions such as cell growth, proliferation, development and oncogenic processes [28]. However, accumulating evidence has suggested a conflicting role for KLF5 in tumorigenesis. On one hand, KLF5 has been demonstrated to be upregulated in some types of human cancers, such as breast and bladder cancer, in which it contributes to tumor progression [29, 30]. On the other hand, KLF5 has been reported to be frequently deleted or downregulated in other kinds of tumors like prostate cancer and acute myeloid leukemia, in which it suppresses cancer cell growth [31, 32]. Thus, KLF5’s function as an oncogene or a tumor suppressor may depend on the types and stages of tumors [33, 34]. More recently, KLF5 was also found to be associated with drug resistance in several tumors. For instance, KLF5 has been reported to strengthen drug resistance in ovarian cancer cells by inducing survivin gene expression [35]. Moreover, KLF5 was a dexamethasone (Dex)-induced gene that contributed to Dex-mediated drug chemoresistance in triple-negative breast cancer [36]. More importantly, a previous study revealed that KLF5 transcriptionally repressed the expression of ATP-binding cassette subfamily G member 2 (ABCG2) and KLF5 knockdown increased the resistance of lung cancer cells to doxorubicin treatment [37]. However, whether KLF5 was involved in hypoxia-induced DDP resistance in NSCLC remains to be explored.

In the present study, we found that KLF5 expression was significantly upregulated in NSCLC cells under a hypoxic condition, consistently with the previous study [38]. Our study further implicated that KLF5 knockdown overturned the cytotoxic effects of DDP and suppressed the expressions of P-gp and HIF-1α in NSCLC cells under hypoxia. Moreover, HIF-1α overexpression abolished KLF5 knockdown-induced suppression of HIF-1α and P-gp expressions in NSCLC cells under hypoxia. KLF5 is well known to act as an upstream regulator of HIF-1α [18]. Additionally, it has been shown that cancer cells develop resistance to chemotherapeutic drugs via hypoxia-induced expressions of drug-pumping proteins, such as P-gp, a well-known transcriptional target of HIF-1 [39]. Accordingly, we concluded that KLF5 knockdown hindered hypoxia-induced DDP resistance via inhibiting P-gp expression in a HIF-1α-dependent manner.

Increased glycolysis is a major causative factor accounting for hypoxia-induced drug resistance in tumors [40]. It has been well accepted that glycolysis is the main energy source for hypoxic cancer cells to produce large amounts of ATP, which enables malignant tumor cells to survive under a hypoxic condition [41]. Therefore, ATP deficiency induced by glycolytic inhibition can enable cancer cells more susceptible to chemotherapeutic drugs [41]. For example, treatment of dichloroacetate (DCA), an inhibitor of the glycolytic pathway, or genetic inhibition of pyruvate dehydrogenase kinase-1 (PDK-1), which is responsible for enhancing glycolysis, attenuated hypoxia-induced resistance to 5-fluorouracil (5-FU) in gastric cancer cells through the alteration of glucose metabolism [23]. Baicalein reversed hypoxia-induced 5-FU resistance in gastric cancer cells through suppression of glycolysis via regulation of the PTEN/Akt/HIF-1α signaling pathway [24]. In the present study, we demonstrated that KLF5 knockdown inhibited hypoxia-induced glycolysis in NSCLC cells, as evidenced by the reduced glucose consumption and lactate production, which was overturned by HIF-1α overexpression. Collectively, these results suggested that KLF5 knockdown inhibited hypoxia-induced DDP resistance by inhibiting HIF-1α-dependent glycolysis.

The PI3K/Akt/mTOR signaling pathway widely distributed in cells has been recognized as an important signaling pathway involved in the regulation of many cellular functions, such as cell proliferation, differentiation, survival, and apoptosis [42]. Abnormal activation of the PI3K/Akt/mTOR pathway is frequently observed in various types of human cancers and therefore targeting this pathway might represent a key therapeutic opportunity for the treatment of tumor [43]. Recent studies show that the PI3K/Akt/mTOR pathway plays an important role in the development of chemotherapy resistance and inhibition of the PI3K/Akt/mTOR pathway reverses drug resistance in several tumors [44, 45]. Emerging evidence suggests that activation of the PI3K/Akt signaling by hypoxia contributes to hypoxia-induced drug resistance in various human cancers [46, 47]. For example, HIF-1α-induced MAX dimerization protein 1 upregulation contributed to hypoxia-induced DDP resistance in osteosarcoma cells through activation of the PI3K/Akt pathway [11]. Wogonin reversed hypoxia-induced multidrug resistance of human colon cancer cells via suppression of HIF-1α and glycolysis, by inhibiting the PI3K/Akt signaling pathway [48]. Similarly, our study proved that KLF5 knockdown inhibited hypoxia-induced activation of the PI3K/Akt/mTOR pathway in NSCLC cells. Besides, KLF5 overexpression promoted hypoxia-induced DDP resistance in NSCLC cells through activation of the PI3K/Akt/mTOR pathway. Based on the above results, we concluded that KLF5 knockdown suppressed hypoxia-induced DDP resistance by inhibiting HIF-1α-dependent glycolysis through inactivation of the PI3K/Akt/mTOR pathway.

## Conclusions

In conclusion, it was demonstrated that KLF5 knockdown showed strong reversal potency of hypoxia-induced DDP resistance in NSCLC cells by inhibiting HIF-1α-dependent glycolysis through inactivation of the PI3K/Akt/mTOR pathway. Therefore, KLF5 may be a novel and promising therapeutic sensitizer to reverse hypoxia-induced DDP resistance in NSCLC cells.

## Abbreviations

KLF5: krüppel-like factor 5
HIF-1α: hypoxia inducible factor-1α
DDP: cisplatin
NSCLC: non-small cell lung cancer cells
P-gp: P-glycoprotein
Akt: protein kinase B
PI3K: phosphoinositide 3-kinase
mTOR: mammalian target of rapamycin
SDS-PAGE: sodium dodecyl sulfate-polyacrylamide gel electrophoresis
DCA: dichloroacetate
PDK-1: pyruvate dehydrogenase kinase-1
